# Wnt/CTNNB1 Signal Transduction Pathway Inhibits the Expression of ZFP36 in Squamous Cell Carcinoma, by Inducing Transcriptional Repressors SNAI1, SLUG and TWIST

**DOI:** 10.3390/ijms21165692

**Published:** 2020-08-08

**Authors:** Emma D. Zanfi, Sebastian Fantini, Roberta Lotti, Matteo Bertesi, Alessandra Marconi, Alexis Grande, Rossella Manfredini, Carlo Pincelli, Tommaso Zanocco-Marani

**Affiliations:** 1 Laboratory of Applied Biology, Department of Life Sciences, University of Modena and Reggio Emilia, 41125 Modena, Italy; emma.zanfi@gmail.com (E.D.Z.); bertesi.matteo@gmail.com (M.B.); alexis.grande@unimore.it (A.G.); 2 Centre for Regenerative Medicine “Stefano Ferrari”, Department of Life Sciences, University of Modena and Reggio Emilia, 41125 Modena, Italy; sebastian.fantini@unimore.it (S.F.); rossella.manfredini@unimore.it (R.M.); 3 Laboratory of Cutaneous Biology, Department of Surgical, Medical, Dental and Morphological Sciences, University of Modena and Reggio Emilia, 41125 Modena, Italy; roberta.lotti@unimore.it (R.L.); alessandra.marconi@unimore.it (A.M.); carlo.pincelli@unimore.it (C.P.)

**Keywords:** *ZFP36*, Wnt/CTNNB1 signaling pathway, squamous cell carcinoma, transcriptional repressors, EMT

## Abstract

The Wnt/CTNNB1 pathway is often deregulated in epithelial tumors. The *ZFP36* gene, encoding the mRNA binding protein Tristetraprolin (TTP), is downregulated in several cancers, where it has been described to behave as a tumor suppressor. By this report, we show that Wnt/CTNNB1 pathway is constitutively activated, and *ZFP36* expression is downregulated in Squamous Cell Carcinoma (SCC) cell lines compared to normal keratinocytes. Moreover, we suggest that the decrease of *ZFP36* expression might depend on the activity of transcriptional repressors SNAI1, SLUG and TWIST, whose expression is induced by Wnt/CTNNB1, highlighting a potential regulatory mechanism underlying *ZFP36* downregulation in epithelial cancers.

## 1. Introduction

*ZFP36* gene encodes the Zinc-finger RNA-binding protein Tristetraprolin (TTP). TTP expression is induced by different stimuli, such as mitogenic agents [1] and inflammation [2,3]. TTP localizes in the nucleus, but stimulation leads to its translocation in the cytosol where it recognizes and binds to specific AU motifs at the 3′UTR (AREs) of its target mRNAs, thereby triggering their degradation [4,5]. Owing to this activity, TTP is involved in the anti-inflammatory response, by targeting and inducing degradation of several pro-inflammatory cytokines mRNAs [6], thereby preventing chronical inflammation [7]. With respect to TTP mediated RNA degradation activity, this protein works most efficiently at a specific concentration, and it is able to self-regulate its own mRNA’s stability in order to remain at optimal levels [8]. TTP’s role in cancer is still controversial. It is recognized as a protein that inhibits tumor progression although, for instance, it also acts on CD8+ lymphocytes by blocking cell-mediated antitumor immune response [9]. *ZFP36* gene is frequently downregulated in different cancers [9], where, although specific mutations affecting it are not described, it seems to act as a tumor suppressor [5,10,11]. In addition, TTP has been shown to be able to revert mesenchymal to epithelial phenotype in many tumor cell lines, by down regulating the transcriptional repressors SNAI1 and TWIST1 [6].

The skin is the largest organ in the human body. To maintain its homeostasis, it relies on keratinocyte stem cells (KSCs), that have potential for self-renewal and differentiation [12,13]. Epithelial differentiation is finely regulated in healthy skin, through a balance between transcription factors, growth factors, structural molecules and enzymes [14,15]. Squamous cell carcinoma (SCC) is the second most frequent non-melanomatous skin cancer, and it develops as a consequence of over-proliferation of epidermal squamous cells [16]. It exists in two forms: cutaneous (cSCC) and mucosal, and originates from mutated keratinocytes (KCs). SCC can have different levels of differentiation, different stages of keratinization and a variable presence of intercellular bridges, depending on the characteristics of the cells of the paving epithelium of the epidermis. This neoplasm can occur as recurrence or de novo on irradiated or chronically damaged skin. KCs are thought to be the “Starting cells” of squamous cell carcinoma [17]. Different cancer stem cells populations have been isolated in SCC; these cells are controlled by the integrin/FAK and the TGF-β signaling pathways. The tyrosine kinase FAK interacts with the Wnt/CTNNB1 signaling pathway in the regulation of the initial stages of skin carcinogenesis [16].

Epithelial to mesenchymal transition (EMT) is a process, in which cells gradually lose the epithelial phenotype assuming a mesenchymal one through changes in gene expression. Cells undergoing EMT tend to lose cell-cell contacts, reshape the cytoskeleton and exponentially increase motility and invasion capacity [18,19,20,21]. When a carcinoma begins to metastasize, cellular changes are the same as in EMT [22]. EMT is driven by a series of transcription factors and proteins involved in Wnt/CTNNB1 pathway such as ZEB1/2, SNAI1/2 (respectively SNAI1 and SLUG) and TWIST1/2 which are activated in the early stages of the transition [19]. Wnt/CTNNB1 signaling, is the canonical pathway of Wnt proteins. An aberrant regulation of this pathway can cause organ fibrosis, degenerative diseases [23], metabolic pathologies and malignancies [24]. CTNNB1 protein (95 kDa) can be divided into three domains: N-terminal domain, armadillo domain with twelve armadillo repeats and the C-terminal domain. Different phosphorylation events mediate CTNNB1 localization [25]. When CTNNB1 is not phosphorylated it tends to accumulate in the cytoplasm. In the nucleus CTNNB1 recognizes and binds the TCF/LEF complex (T Cell Factor/Lymphoid Enhancer-binding Factor), a transcription factor which recognizes WREs (Wnt-Response-Elements) on Wnt target genes. In vitro, the transcriptional complex is able to bind the promoter region of the E-cadherin gene, leading to its down-regulation [26,27]. Moreover TCF/LEF bound to CTNNB1 stimulates transcription of Wnt target genes, such as TWIST and SNAI1 [28]. Consequently, Wnt/CTNNB1 pathway mediates adhesion between cells, their mobility, and proliferation [29]. During EMT, SNAI1 and SLUG (SNAI2) induce the expression of mesenchymal genes or repress epithelial ones by binding E-box sequences via their DNA binding domain. TWIST proteins are bHLH (basic helix-loop-helix) transcription factors with a comparable activity [6]. TTP is able of inhibiting EMT, by directly binding ARE sequences located in the mRNAs of TWIST1 and SNAI1, thereby leading to their degradation [30]. By this paper, we suggest the existence of an inverse correlation between TTP and the transcriptional repressors SNAI1, SLUG and TWIST, in that not only they are directly targeted by TTP, but they are capable of transcriptionally repressing the expression of the *ZFP36* gene.

## 2. Results

### 2.1. Wnt/CTNNB1 Pathway Constitutive Activation and ZFP36 Down-Regulation in SCC Cell Lines Compared to Normal Keratinocytes

**Figure 1:**
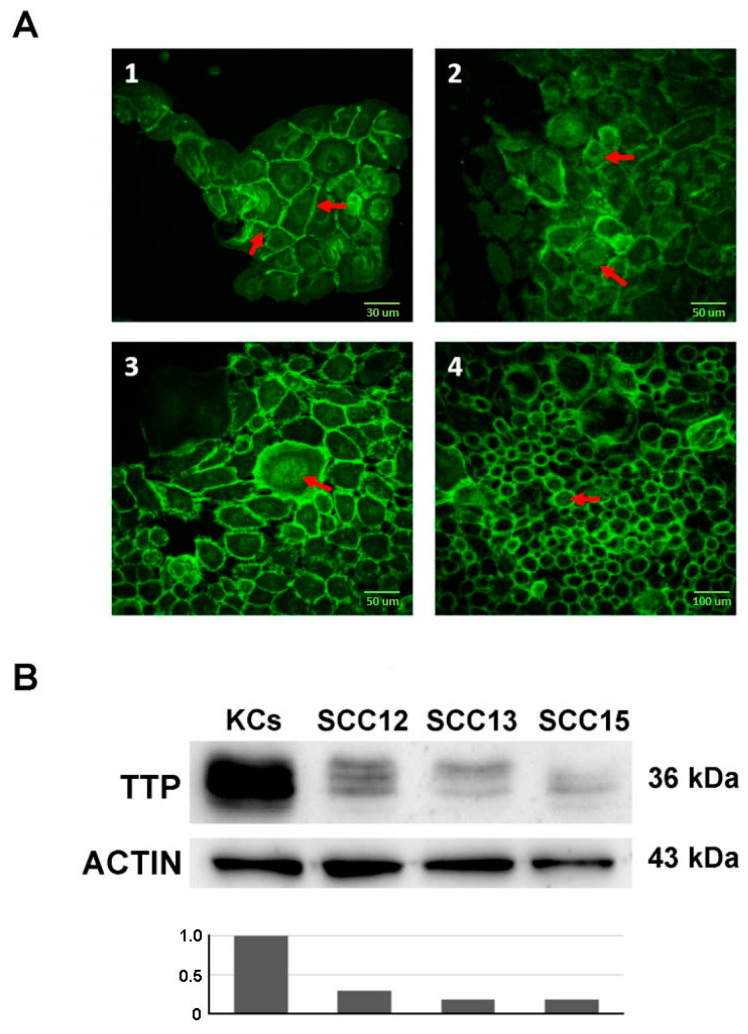
Immunofluorescence highlighting CTNNB1 localization and immunoblotting of ZFP36 expression. Panel (**A**) shows confocal microscope images of healthy keratinocytes (panel (**A**(**1**))) and SCC12, SCC13 and SCC15 (Panels (**A**(**2**)), (**A**(**3**)) and (**A**(**4**)) respectively). Scale bars 30 µm, 50 µm and 100 µm. Panel (**B**) shows the western blot analysis of TTP expression in the three SCC cell lines compared to normal keratinocytes. The plot below indicates the normalized densitometric analysis of immunoblot.

CTNNB1 subcellular localization was assessed by immunofluorescence. As shown in Figure 1A(1), in healthy keratinocytes CTNNB1 is found predominantly in cell-cell junctions and in the cytoplasmatic membrane. In SCC cell lines (Figure 1A(2–4)), CTNNB1 shows perinuclear localization, as shown by fluorescence signal in the cytoplasm around the cells’ nucleus. Among the three analyzed cell lines, perinuclear localization is observed principally in the SCC13 and SCC15 lines (Figure 1A(2) and A(3) respectively), and less strongly in SCC12 cells (Figure 1A(4)), that display a distribution of CTNNB1 more similar to healthy keratinocytes. Figure 1B shows a western blot analysis suggesting that TTP expression is downregulated in SCC12, SCC13 and SCC15 cell lines compared to normal keratinocytes.

### 2.2. Expression of SLUG, SNAI1 and TWIST in SCC12, 13 and 15 Cell Lines Compared to Normal Keratinocytes

**Figure 2:**
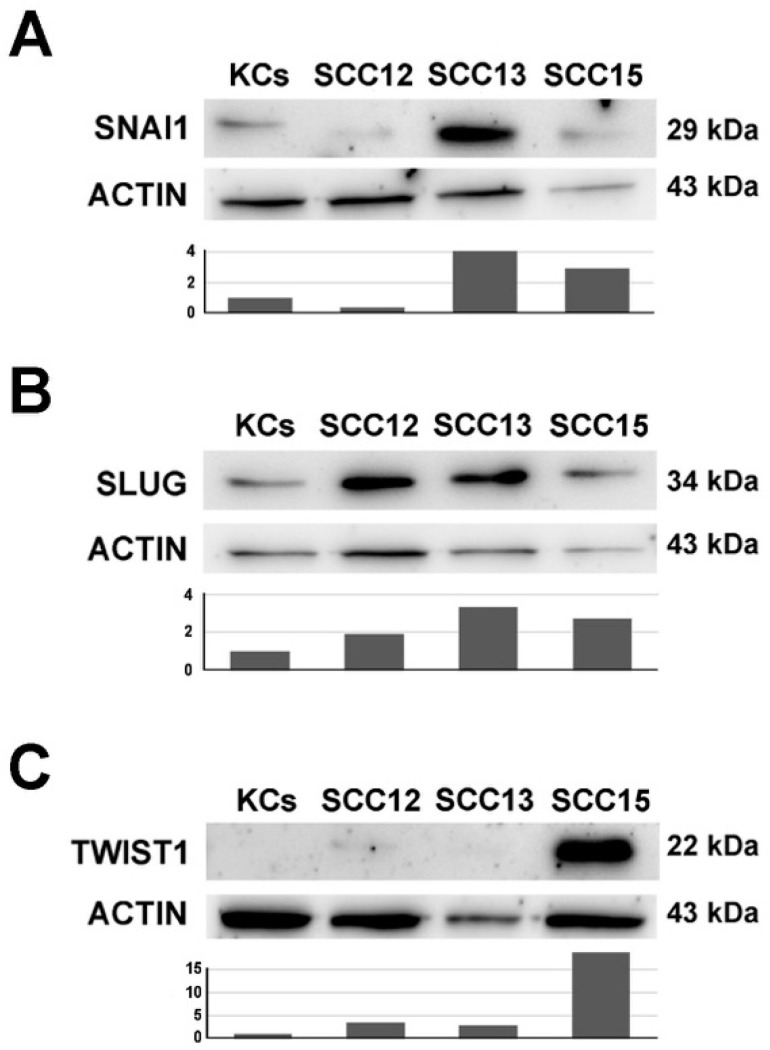
Immunoblotting of SNAI1 (Panel **A**), SLUG (Panel **B**) and TWIST (Panel **C**) proteins expression in healthy keratinocytes and SCC cell lines (29 kDa, 34 kDa and 22 kDa respectively). The plot below indicates the normalized densitometric analysis of immunoblot.

Figure 2 depicts a western blot analysis of the transcriptional repressors SLUG, SNAI1 and TWIST (Figure 2A, B and C, respectively) in three SCC cell lines compared to normal keratinocytes. The three repressors are expressed at different levels in the three cell lines. SLUG protein is upregulated predominantly in SCC13 and to a lesser extent in SCC15 and SCC12 cells. SNAI1 protein is induced especially in SCC13 and SCC15 cells and is almost completely absent in SCC12. TWIST protein is only upregulated in SCC15 cells, compared to normal keratinocytes. Overall, at least one of the three repressors is induced in every SCC cell line compared to healthy keratinocytes.

### 2.3. Treatment with the Wnt/CTNNB1 Inhibitor FH535 Induces ZFP36 Up-Regulation and the Downregulation of Transcriptional Repressors SNAI1, SLUG and TWIST

**Figure 3:**
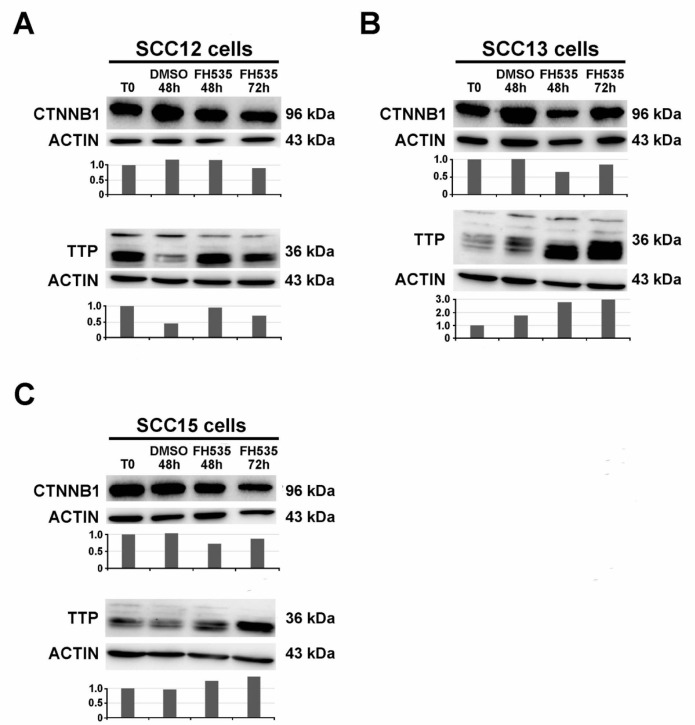
CTNNB1 (96 kDa) and TTP (36 kDa) expression in SCC cell lines treated with FH535, inhibitor of Wnt/CTNNB1 signal pathway. T0 refers to the beginning of the treatment with FH535 or DMSO, respectively. Analysis of the treatment was performed at 48 and 72 h by Western Blot. In the panels (**A**–**C**) show expression of CTNNB1 and TTP in SCC12, SCC13 and SCC15 respectively. The plot below indicates the normalized densitometric analysis of immunoblot.

**Figure 4:**
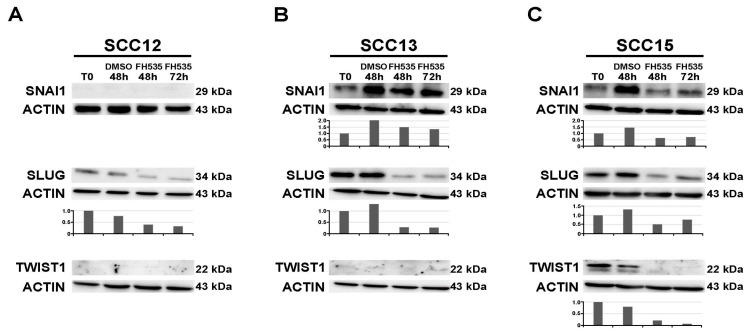
Western blot analysis of SNAI1, SLUG and TWIST expression (29 kDa, 34 kDa and 22 kDa respectively) in SCC cell lines treated with FH535. T0 refers to the beginning of the treatment with FH535 or DMSO. Panels (**A**–**C**) show repressors’ expression following treatment in SCC12, SCC13 and SCC15 cells respectively. The plot below indicates the normalized densitometric analysis of immunoblot.

Western blot analysis was performed 48 and 72 h upon treatment with FH535 in the cell lines SCC12, SCC13 and SCC15. Following FH535 treatment, the expression of CTNNB1 tends to decrease, while *ZFP36* is upregulated in the three cell lines (Figure 3A–C). Figure 4A–C show a western blot analyzing the expression of repressors SNAI1, SLUG and TWIST in SCC12, SCC13 and SCC15 cell lines (panels A, B, C, respectively). SCC12 cells express neither SNAI1 nor TWIST. They only show SLUG expression, which is downregulated following treatment with the FH535 inhibitor as represented in Figure 4A. SCC13 cells show SNAI1 and SLUG expression. These two repressors are both down-regulated following treatment with FH535 (Figure 4B). SCC13 do not show TWIST expression (Figure 4B). Figure 4C shows the expression of the three transcriptional repressors in the SCC15 cell line. As in the previous tumor lines, SLUG and SNAI1 are downregulated by the treatment with FH535. Moreover, in this cell line TWIST follows the same path and it shows complete downregulation at 72 h after treatment.

### 2.4. SNAI1 and SLUG Proteins Bind the ZFP36 Promoter Region Both in Healthy Keratinocytes and in SCC15 Cell Line

**Figure 5:**
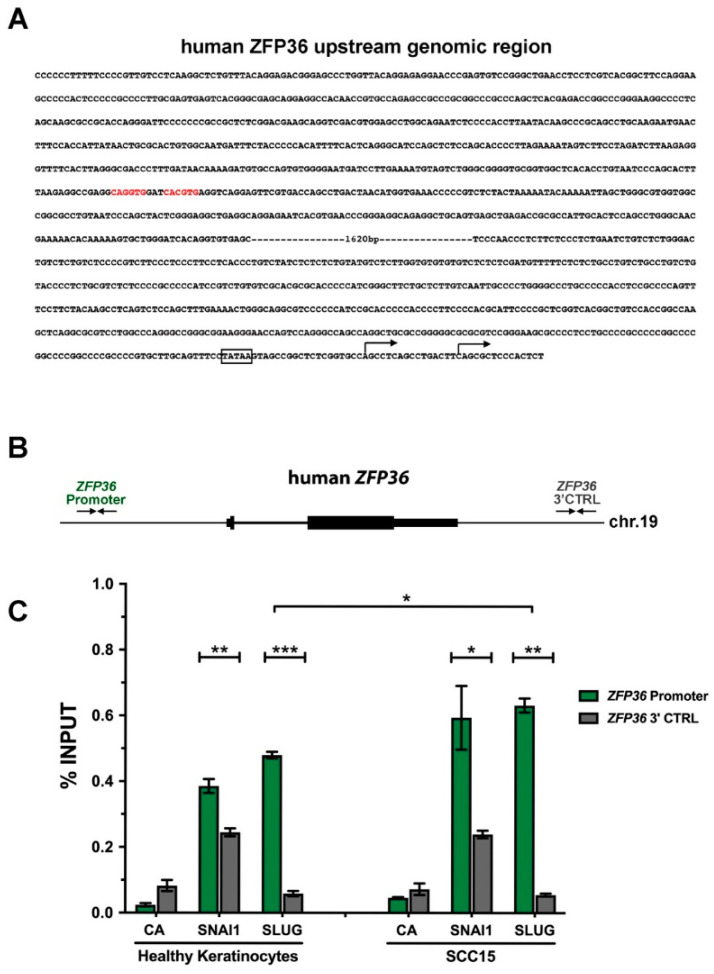
(**A**) Sequence of the genomic region located upstream of the human *ZFP36* gene, and corresponding to the putative promoter region, is reported in bold. Two putative E-Box elements (5′-CANNTG-3′) were indicated in red. A canonical TATA box element was highlighted by box. Two alternative transcriptional start sites (TSS) were indicated by black arrows. (**B**) Schematic representation of the human genomic region encompassing the *ZFP36* locus. Inward facing arrows display the primer set amplifying the ZFP36 promoter region (*ZFP36* Promoter) and a negative control region located downstream from the *ZFP36* gene (*ZFP36* 3′CTRL). (**C**) Chromatin immunoprecipitations were performed with anti-SNAI1 and anti-SLUG antibodies. CA = control antibody. Green bars represent the binding on the *ZFP36* promoter region whereas dark grey columns indicate the enrichment on its relative control region. Data are expressed as mean percentage of total input chromatin ± SEM of at least three independent experiments. * *p*-value ≤ 0.05; ** *p*-value ≤ 0.01; *** *p*-value ≤ 0.001 evaluated with *t*-test.

To characterize the *ZFP36* promoter, and to verify a possible regulation of its expression by the above mentioned transcriptional repressors, we performed an in silico analysis of 2994 base-pairs’ (bp) genomic region upstream of the human *ZFP36* gene. To this purpose E-box sequences (5′-CANNTG-3′), known binding sites for SNAI1, SLUG and TWIST proteins, were searched using MatInspector software (Genomatix). We discovered a nearly canonical E-Box element, composed by two distinct and adjacent 5′-CANNTG-3′ sequences, located 2416 bp upstream from the most common annotated transcriptional start site (TSS1) of *ZFP36*. Moreover, a TATA-box regulatory sequences was annotated 24 nt upstream from the TSS1. A second, alternative TSS (TSS2) has been mapped 16 nt downstream from TSS1 (Figure 5A).

To test whether this E-Box could indeed bind to these transcriptional repressors, we performed chromatin immunoprecipitation assay in healthy keratinocytes and SCC15, unique among the previously exploited squamous cancer cell lines that simultaneously expresses all the three mentioned repressors (as shown in Figure 2). To date, both SNAI1 and SLUG proteins were found to bind significatively to the E-Box within the *ZFP36* promoter when compared to a negative control region located downstream to the *ZFP36* gene both in human keratinocytes and in SCC15 cells (Figure 5B,C). In addition, the binding of SLUG to the *ZFP36* promoter in SCC15 cells is significantly higher than in healthy keratinocytes although no obvious alteration in its expression was observed (Figure 2). No significant differences were instead observed between the binding capability of SNAI1 to *ZFP36* promoter in healthy keratinocytes and SCC15 cell line (Figure 5C).

Together, these data hence demonstrate that SNAI1 and SLUG repressors actually bind to *ZFP36* proximal putative promoter region and hence might be involved in its transcriptional repression.

## 3. Discussion

Although *ZFP36* expression is lost in several malignancies, the mechanisms underlying this event are still poorly understood. Specific mutations in the promoter region of *ZFP36* are not been described so far. Epigenetic causes have been proposed [24]. However, we hypothesize that, since, in general, loss of *ZFP36* fosters tumor progression, in different cell contexts *ZFP36* down-regulation might result from the modulation of pathways that are crucial to a specific tumor. To this regard, we observed in a previous work that *ZFP36* expression in colorectal carcinoma cell lines is inversely correlated to Wnt/CTNNB1 pathway activity [6]. Recently, it has also been suggested that miRNA-423 is capable of promoting tumor cell proliferation and migration by enhancing Wnt/CTNNB1 activity, thereby inducing *ZFP36* down-regulation [31]. We also analyzed the activity toward *ZFP36* promoter of specific microRNAs induced by Wnt/CTNNB1, among others (microRNA29b1/a), but never get conclusive results (data not shown). Moreover, we were able to exclude the possibility that TCF7L2/CTNNB1 transcriptional complex might directly bind *ZFP36* promoter, thereby repressing its expression (data not shown). The correlation between Wnt/CTNNB1 and *ZFP36* is noteworthy and the molecular process that links the signal transduction pathway to *ZFP36* modulation deserves to be clarified. The aim of this work is to demonstrate that the correlation existing between *ZFP36* and the Wnt/CTNNB1 signal transduction pathway is mediated by three transcriptional repressors (SNAI1, SLUG and TWIST) that are induced by CTNNB1 pathway and that modulate *ZFP36* by binding to its promoter.

The protein product of *ZFP36* (TTP) is known to directly target the mRNAs encoding SNAI1, SLUG and TWIST [6]. When Wnt/CTNNB1 signaling is deregulated, the sustained expression of SNAI1, SLUG and TWIST is involved in the epithelial to mesenchymal transition. Since it is known that *ZFP36* is downregulated in epithelial cancer, that its down-regulation stabilizes the expression of a set of genes involved in EMT and that restoration of its expression contributes to the rescue of a more epithelial phenotype [6], the existence of this functional inverse correlation between TTP and the repressors made it plausible to hypothesize that the repressors might be the link allowing Wnt/CTNNB1 signaling to negatively regulate *ZFP36* gene.

We elected to perform this study on a model of epithelial cancer and chose SCC since, to date, there were no data on this disease and *ZFP36*. As we observed by immunofluorescence, Wnt/CTNNB1 is constitutively active in the cell lines SCC12, SCC13 and SCC15. Moreover, we observed that *ZFP36* is down-regulated in these cells compared to healthy keratinocytes, as opposed to transcriptional repressors SNAI1, SLUG and TWIST, that are upregulated in these SCC lines as a consequence of Wnt/CTNNB1 constitutive activity. We observed that inhibition of Wnt/CTNNB1 via the specific molecule FH535 determines a decrease of SNAI1, SLUG and TWIST expression and the rescue of *ZFP36* expression. Western blots throughout the paper show the presence of multiple differently migrating bands for TTP. In our opinion these bands are specific and their different height represents a different phosphorylation level. In fact, it is well known that TTP can be phosphorylated by p38 MAP kinase and that this event results in protein stabilization and in the loss of mRNA destabilizing activity [32]. Moreover, as mentioned in the introduction, TTP mRNA destabilizing capability is active at a specific protein concentration [8]. At different concentrations the protein undergoes inactivation by phosphorylation. In our hands this makes over-expression experiments particularly tricky, since the achievement of very high expression levels often leads to the accumulation of an inactive form of TTP.

In order to further verify our hypothesis, we analyzed by chromatin immunoprecipitation the ability of the transcriptional repressors SNAI1 and SLUG to bind *ZFP36* promoter at specific E-boxes. Results showed that both repressors are indeed capable of binding *ZFP36* promoter and, in particular, the binding of SLUG to the promoter is significantly higher in SCC15 cells than in healthy keratinocytes. Results obtained on SNAI1, although statistically not significant, suggest that also this repressor preferentially binds *ZFP36* promoter in SCC15 cells compared to healthy keratinocytes. A wider set of chromatin immunoprecipitation data is needed to confirm statistical significance. Chromatin immunoprecipitation assays on TWIST binding are currently ongoing.

In general, we showed that in SCC, and possibly in other tumors of epithelial origin characterized by EMT, where *ZFP36* is down-regulated and the alteration of Wnt/CTNNB1 signaling plays a major role, the link between the signal transduction pathway and *ZFP36* is represented by the transcriptional repressors described in this paper. In tumors of different origin, where Wnt/CTNNB1 signaling is not involved and *ZFP36* is down-regulated, in the absence of specific mutations or epigenetic events (that are not to be excluded), the tumor advantage-conferring TTP silencing can be achieved as a consequence of the deregulated activity of other specific pathways. This idea seems also acceptable considering the fact that TTP expression is often induced physiologically in order to switch off a biological process, be it the inflammatory response, proliferation or other. Conversely, different deregulated pathways underlying transformation and the origin of different tumors might converge, among many other consequences, to the inactivation of the expression of the *ZFP36* gene.

## 4. Materials and Methods

### 4.1. Cell Cultures, Treatments and Transfections

SCC12, SCC13, and SCC15 cell lines were purchased from ATCC (Teddington, UK) and maintained in SCC medium (45% DMEM, 45% Ham’s F12, 10% fetal bovine serum, 2% L-Glutammine, 1% penicillin/streptomycin/amphotericin, 1 M HEPES, and 400 ng/mL hydrocortisone), under standard conditions 37 °C, 5% CO_2_. Normal human keratinocytes were isolated from healthy skin biopsies obtained from waste materials from the surgical room, after the patient’s informed consent signature, in Policlinico di Modena - University Hospital of Modena, as previously reported [33]. They were grown in Keratinocyte Growth Medium (KGM; Lonza, Walkersville, MD, USA).

The tumor lines were treated with FH535 (Sigma, St. Louis, Missouri, USA) dissolved in DMSO and diluted in culture medium at the concentration of 30 μM. Control cells received the same volume of DMSO [6].

### 4.2. Western Blot Analysis

Total protein extracts were obtained by lysing the cells in RIPA buffer (50 mM TRIS-HCl pH 7.4, 150 mM NaCl, 1% NP40, 1 mM sodium deoxycholate, 1 mM sodium orthovanadate, 1 mM EDTA) with addition of Protease Inhibitor Cocktail 1x (Roche Applied Science, Penzberg, Germany). Cells were resuspended and centrifuged in the buffer and subsequently quantified by Bradford assay. A fixed amount of protein for each sample was loaded on SDS-Polyacrylamide gels for each sample and transferred to a nitrocellulose membrane. The membranes were blocked with 5% non-fat milk in TBS + 0.1% Tween, and then immunoblotted overnight at 4 °C with the following primary antibodies: Tristetraprolin (#71632, Cell Signalling Technologies, Danvers, MA, USA); TWIST1 (#46702, Cell Signalling Technologies, Danvers, MA, USA); SLUG (#9585, Cell Signalling Technologies, Danvers, MA, USA); SNAI1 (#3895, Cell Signalling Technologies, Danvers, MA, USA). One-hour incubation at room temperature was enough for the primary antibody against CTNNB1 (BD Transduction Laboratories 610154, South San Francisco, California, USA). The nitrocellulose membranes were then incubated with secondary antibodies: either Goat anti-rabbit IgG-HRP or Goat anti-mouse IgG-HRP (Santa Cruz Biotechnology sc-2004 and sc-2005, Santa Cruz, CA, USA). Chemiluminescence was detected with ChemiDoc (BIO-RAD, Hercules, California, USA).

### 4.3. Immunofluorescence

Untreated cells were grown on slides coated with 1 mL of collagen IV and fixed with 4% paraformaldehyde for 20 min at room temperature, then washed with PBS. Cells were permeated with 1% Triton X100 for 5 min on ice, and then incubated with Blocking Solution (Goat Serum 10% and BSA 1% in PBS) for 20 min. The slides were incubated with the primary antibody anti-CTNNB1 (BD Transduction Laboratories 610154) diluted 1:100 in blocking solution, overnight at 4 °C in humidified chamber. Subsequently the slides were incubated for 45 min at room temperature with anti-mouse Alexa Flour 488 antibody (Invitrogen, Carlsbad, California, USA), diluted 1:300. Fluorescent samples were analyzed by confocal scanning laser microscope (Leica TCS SP2, Wetzlar, Germany).

### 4.4. Chromatin Immunoprecipitation

Chromatin immunoprecipitation assays were performed as previously described [34]. Briefly, chromatin from human keratinocytes and human SCC15 cell line was crosslinked with 1% formaldehyde and subjected to immunoprecipitation with 5 μg anti-SNAI1 (AF3639, R&D Systems, Minneapolis, Minnesota, USA), 5 μg anti-SLUG (C19G7) (9598S, Cell Signalling Technologies, Danvers, MA, USA) whereas 5 μg of normal mouse IgG (sc-2025, Santa Cruz Biotechnology, Santa Cruz, CA, USA) were used as control antibody (CA). The enrichment of the amplified genomic region in immunoprecipitates was evaluated by quantitative real-time PCR (qPCR) and calculated as a percentage of total input chromatin. Mean enrichments for each ChIP were assessed using three independent experiments. Primers used in qPCR analysis were: ZFP36-Prom Fw_: 5′-AGGGTTTTCACTTAGGGCGA-3′; ZFP36-Prom Rev_: 5′-TTAGTCAGGCTGGTCACGAA-3′; ZFP36-3′CTRL Fw_: 5′-GCTCCTTGACCCAGCTTCGG-3′; ZFP36-3′CTRL Rev_: 5′-ACATTCCGTCCTCCCCACCC-3′. Statistical analysis (t-test) was performed with Prism GraphPad version 8.0, San Diego, CA, USA [34].

## Abbreviations

ARE	AU-rich element
ATCC	American Type Culture Collection
bHLH	basic Helix-Loop-Helix
bp	base pairs
cSCC	cutaneus Squamous Cell Carcinoma
CTNNB1	β-catenin
DMEM	Dulbecco’s Modified Eagle Medium
DMSO	Dimethyl sulfoxide
EDTA	Ethylenediaminetetraacetic acid
EMT	Epithelial to Mesenchymal Transition
FAK	Focal Adhesion Kinase
FH535	C13H10Cl2N2O4S
Ham’s	F12 Ham’s Nutrient Mixture F12
HEPES	4-(2-hydroxyethyl)-1-piperazineethanesulfonic acid
HRP	Horseradish peroxidase
KSC	Keratinocyte Stem Cells
PBS	Phosphate-Buffered Saline Phosphate-Buffered Saline
PCR	Polymerase Chain Reaction
RIPA	Radioimmunoprecipitation Assay
SDS	Sodium Dodecyl Sulfate
SEM	Standard Error of the Mean
SCC	Squamous Cell Carcinoma
TBST	Tris-buffered saline, 0.1% Tween 20
TCF/LEF	T Cell Factor/Lymphoid Enhancer-binding Factor
TGF-β	Transforming Growth Factor β
TTP	Tristetraprolin
UTR	Un-Traslated Region
WREs	Wnt-Response-Element
ZFP36	Zinc Finger Protein of 36 kDa
